# A 12-month prospective real-life study of opicapone efficacy and tolerability in Emirati and non-White subjects with Parkinson’s disease based in United Arab Emirates

**DOI:** 10.1007/s00702-023-02700-y

**Published:** 2023-10-05

**Authors:** Vinod Metta, Huzaifa Ibrahim, Neha Muralidharan, Kislyn Rodriguez, Therese Masagnay, Judith Mohan, Arlet Lacsina, Abdullah Ahmed, Hani T. S. Benamer, Guy Chung-Faye, Rukmini Mrudula, Cristian Falup-Pecurariu, Carmen Rodriguez-Blazquez, Rupam Borgohain, Vinay Goyal, Kalyan Bhattacharya, K. Ray Chaudhuri

**Affiliations:** 1 King’s College London, Department of Neurosciences, Institute of Psychiatry, Psychology and Neuroscience and Parkinson’s Foundation Centre of Excellence, King’s College Hospital, London, UK; 2 Parkinson’s Foundation Centre of Excellence, King’s College Hospital, Dubai, United Arab Emirates; 3 Parkinson’s Association United Arab Emirates, Dubai, United Arab Emirates; 4 College of Medicine, Mohammed Bin Rashid University of Medicine and Health Sciences, Dubai, United Arab Emirates; 5 Nizams Institute of Medical Sciences, Hyderabad, Telangana India; 6 Department of Neurology, Transilvania University of Brasov, Brasov, Romania; 7 Institute of Salud Carlos 111, Madrid, Spain; 8 Institute of Movement Disorders and Parkinson’s Centre, Medanta Hospitals, Delhi, India; 9 R G Kar Medical College and Hospital, Kolkata, India

**Keywords:** Catechol-O-methyltransferase, Entacapone, Opicapone, Nonmotor symptoms

## Abstract

Parkinson’s disease (PD) is the second most common neurodegenerative disorder, and the condition is complicated by the emergence of wearing off/motor fluctuations with levodopa treatment after a variable period. COMT inhibitors when used as adjunct therapy to levodopa tend to smoothen out these wearing off fluctuations by enhancing delivery of levodopa and increasing its bioavailability to the brain. The study was conducted to investigate the motor and nonmotor effect, safety and tolerability of the third generation once-daily COMT inhibitor (opicapone), as add-on, adjuvant therapy to levodopa and at 6 and 12 months follow-up in a real-life cohort of consecutive Emirati and non-White PD patients. A real-life observational analysis using tolerability parameters as used previously by Rizos et al. and Shulman et al. based on clinical database of cases rat Kings College Hospital Dubai Parkinson care database. This was a prospective, single-arm follow-up clinical evaluation study that evaluated the effectiveness of opicapone 50 mg once-daily regime in 50 patients diagnosed with idiopathic neurodegenerative disorder. All patients were assessed with scales used in clinical pathway and include motor Unified Parkinson’s Disease Rating Scale (UPDRS), nonmotor symptom scale (NMSS), quality of life (PDQ8) Parkinson’s fatigue scale (PFS16) and King’s Parkinson’s Pain Scale (KIPS). Out of 50 patients treated with opicapone (72% male, mean age 66.9 years (SD 9.9, range 41–82 years) and mean duration of disease 5.7 years (SD 2.5 range (2–11), there was significant statistical improvements shown in motor function-UPDRS part 3: baseline 40.64 ± 2.7, at 6 months 32.12 ± 3.14 and after 12 months 33.72 ± 3.76. Nonmotor burden NMSS: 107.00 ± 21.86, at 6 months 100.78 ± 17.28 and 12 months 96.88 ± 16.11. Reduction in dyskinesias (UPDRS part 4): baseline 8.78 ± 1.07, at 6 months 7.4 ± 0.81 and 12 months 6.82 ± 0.75. Opicapone provides beneficial motor and nonmotor effects in Emirati and other non-White Parkinson’s patients, resident in UAE, proving its efficacy across different racial groups as COMT activity may vary between races.

## Introduction

Parkinson’s disease (PD) is the second most common neurodegenerative disorder,# with an increasing prevalence with age (Lau and Breteler 2006) In the Middle Eastern countries, the prevalence of PD ranges from 31.4 to 557.4 per 100,000 (Alamri et al. 2015). A recent study by Metta et al. highlights heterogenetic and endophenotype variations of Parkinson’s disease in the UAE population and the importance of prompt diagnosis and dopaminergic dose optimisation (EmPark study) (Metta et al. 2022). Opicapone (OPC) is a once-daily, potent third-generation longer-acting catechol-O-methyltransferase (COMT) inhibitor that is generally well tolerated, efficacious, and has a favourable safety profile. Based on two pivotal clinical trials, BIPARK-I and II, OPC was first approved in the European Union countries as an adjunctive therapy to levodopa preparations and has currently been approved and marketed in the USA, Japan, South Korea, Australia and other countries (Ferreira et al. 2016; Lees et al. 2017). A significant beneficial effect of opicapone in reducing motor ‘off’ episodes duration and severity has been recently reported in the BIPARK I and BIPARK II studies (Ferreira et al. 2016; Lees et al. 2017). The BIPARK II study also reported a positive signal nonmotor symptoms of sleep and fatigue as assessed by the nonmotor symptoms scale (NMSS) (Lees et al. 2017; Hauser et al. 2020; Oliveira et al. 2015). Another European study (OPTIPARK study), a single-arm, prospective, open-label trial conducted in Germany and the UK has also showed a significant reduction in the NMSS score in PD patients, who were treated with opicapone after 3 months follow-up (Reichmann et al. 2020). In this study, we report real-life tolerability of opicapone in a non-White and Emirati PD patients residing in United Arab Emirates and attending a bespoke movement disorders care clinic. We believe this is the first study addressing the motor and nonmotor effects of adding opicapone in a non-White PD population.

## Methods

### Study design

This was a real-life observational analysis using tolerability parameters as used previously by Rizos et al. (Rizos et al. 2020) and Forbes et al. (Forbes et al. 2003) based on clinical database of cases recorded at Kings College Hospital Dubai.

Fifty consecutive non-White expat subjects and Emirati subjects with PD taking opicapone were included in the study at King’s College Hospital London, Dubai. Opicapone was made available to the patients as part of UAE national law approved compassionate usage programme (CU Ref KHQ/PO/2874)). A CU is defined by the World Health Organization as a programme that is intended to provide potentially life-saving treatments to patients suffering from a disease for which no satisfactory authorized therapy exists and/or who cannot enter a clinical trial, or use drugs that are approved in one country but not available globally. In UAE, CUs are regulated by the Ministry of Health and Prevention (MOHAP) according to the Health Regulatory Act that allows import of small quantities of new drugs by an autonomous medical institution for the treatment of patients suffering from life-threatening diseases or diseases causing serious permanent disability, or diseases requiring therapies for unmet medical needs. Patients already being treated with COMT inhibitors (ex entacapone) or in combination (levodopa, carbidopa, entacapone) or with severe hepatic impairment, any contraindications to COMT inhibitors or any other concomitant neurodegenerative diseases were excluded from this study.

### Institutional review board statement

This study was carried out in accordance with the local ethical committee guidelines. Prior to participating in the study, all patients provided written consent and all data were stored in an anonymized fashion in accordance with the ongoing UK portfolio adopted by the NILS longitudinal cohort study at the National Parkinson’s Centre of Excellence at Kings College Hospital in London, Dubai, in accordance with the General Data Protection Regulation (GDPR UAE). The NILS (UK) study has been authorized by local ethics committees (NRES South-East London REC3, 10,084, 10/H0808/141).

### Patient selection

Patients with a confirmed diagnosis of Parkinson’s disease (PD) who met the UK PD Brain Bank criteria were recruited. Referrals to national Parkinson’s Centre of Excellence Kings College Hospital, Dubai, from all over the UAE (mainly from Dubai, Abu Dhabi, Sharjah, Al Ain, Ras Al Khaimah and others) and self-referrals were included. PD patients already on levodopa treatment received opicapone 50 mg once daily (OD) at least 1 h before start or end of daily dose of levodopa.

## Informed consent

Informed consent was obtained from patients/carers/all participants involved in this study.

### Assessments (baseline and 6 and 12 months follow-up)

During the consultation, as a part of good clinical practice, standardized assessment protocols such as the demographics, age, gender and disease duration, were used, as well as levodopa equivalent daily dose calculation (LEDD) (Tomlinson et al. 2010) and other scales like Hoehn and Yahr staging (H&Y) (Hoehn et al. 1998) and NMSS were administered. The NMSS is a rater-administered method of comprehensive assessment of non-motor symptoms in PD patients, including 30 items grouped in nine relevant domains: (1) cardiovascular including falls, (2) sleep/fatigue, (3) mood/apathy, (4) perceptual problems/hallucinations, (5) attention/memory, (6) gastrointestinal tract, (7) urinary function, (8) sexual function, and (9) miscellaneous. The NMSS score for each item is based on multiple scores of severity (from 0 to 3) and frequency (from 1 to 4) (Ray Chaudhuri et al. 2013). Parkinson’s Disease Questionnaire-8 (PDQ-8) is a specific instrument for assessment of health-related quality of life in PD (Borges 2005), PD Sleep Scale version (PDSS), a 15-item, patient- completed clinical tool, was used to assess the frequency of sleep disturbances during the past week in PD patients (Chaudhuri et al. 2002). MMSE (Mini-Mental State Examination) (Folstein et al. 1975) and PFS 16 (Parkinson’s Fatigue Scale) (Brown et al. 2005) were also used. Furthermore, we included data from patient-reported outcomes, i.e., Hospital Anxiety and Depression Scale (HADS-total), a 14-item, patient-completed scale with subscales for anxiety (HADS-A) and depression (HADS-B) (Stern 2014) was applied. Details of these validated scales have been published elsewhere and the assessments were performed in line with the NILS assessment, a national study by the National Institute of Health Research in the UK (UKCRN No: 10,084) currently containing data of over 1600 PD patients.

### Statistical methods

No sample size was estimated for this study. All participants fulfilling the inclusion criteria within the study period were included in the study. Data were analysed using SPSS 22.0 for Windows. Continuous variables were expressed as the mean ± SD. Analyses were descriptive for the primary and secondary outcome measures and mean changes from baseline to 6 months and 12 months follow- up were analysed using Wilcoxon signed rank test. A *p* value less than 0.05 was considered as statistical significance.

## Results

Out of 50 patients (35 non-White and 15 Emirati) treated with opicapone, 72% showed male preponderance (Table 1). The mean age of the study subjects was 66.9 years (standard deviation: 9.9), age ranged from 41 to 82 years (SD 9.95) and mean duration of disease was 5.7 years with SD 2.5.

**Table 1:** Demographic and baseline clinical characteristics of the study population

Variables	Range	Mean ± SD
Age in years	41–82	66.98 ± 9.95
PD duration in years	2–11	5.74 ± 2.50

Variables	*N*	%
*Sex*		
Female	14	28.0
Male	36	72.0
*Origin*		
Emirati	15	30.0
NonEmirati	35	70.0
*Type of Parkinson’s disease*		
AKD	26	52.0
APD	1	2.0
Mixed	6	12.0
TD	14	28.0
YOPD/AKD	2	4.0
YOPD/DDS/AKD	1	2.0
*HY stage*		
1.5	2	4.0
2.0	6	12.0
2.5	19	38.0
3.0	19	38.0
3.5	3	6.0
4.0	1	2.0
*Opicapone*		
50 E	29	58.0
50 S	21	42.0

*SD*  standard deviation, *AKD* akinetic dominant, *APD* Advanced Parkinson’s disease, *TD* tremor dominant, *YOPD* Young-onset Parkinson’s disease, *DDS* dopamine dysregulation syndrome, opicapone was administered as a once-daily regimen either at start (S) of levodopa or at end (E) of levodopa

**Fig. 1:**
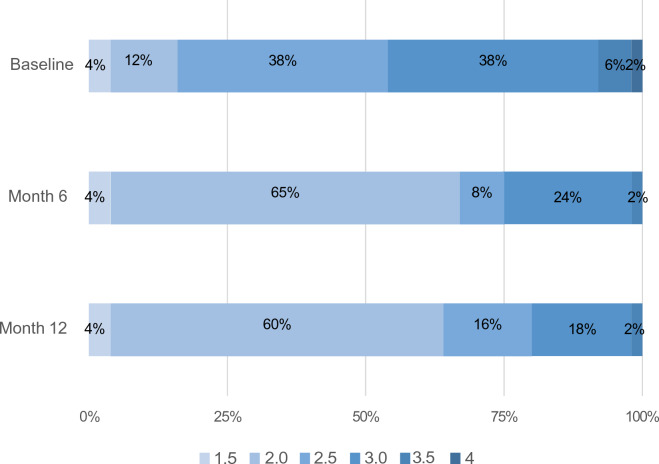
Hoehm and Yahr stage distribution at baseline distribution and at follow-up

At baseline, the majority of the patients (38%) had Hoehn and Yahr (HY, Fig. 1) stage 2.5 with predominant (52%) akinetic dominant PD. 58% of the patients were taking opicapone at the end of the levodopa dose and 42% at the start of the levodopa regime.

### Chi-square test for baseline vs month 12 scores

At baseline, the mean MDS-UPDRS part 3 score (Table 2) was 40.64 ± 2.7, which after opicapone administration significantly (*p* < 0.001) reduced after 6 months (32.12 ± 3.14) and 12 months (33.72 ± 3.76). The most striking feature in our study was that post-6 and 12 months opicapone treatment, there was notable reduction in MDS-UPDRS part 4, which included the items duration, disability and painful dyskinesias at baseline (8.78 ± 1.07) that significantly reduced (*p* < 0.001) following 6 months (7.4 ± 0.81) 12 months (6.82 ± 0.75), respectively. Additionally, NMSS total score, HADS, PFS 16, KPPS score as well as PDQ 8 showed significant improvement, while PDSS showed a significant improvement at 6 months (Table 2).

**Table 2:** Comparison of clinical variables at baseline and follow-up

Variable	Baseline Mean ± SD	Month 6 Mean ± SD	_p_a	Month 12 Mean ± SD	_p_b
Current LEDD	753.40 ± 233.09	713.40 ± 219.27	0.001	641.40 ± 196.56	< 0.001
MDS-UPDRS part 3	40.64 ± 2.77	35.12 ± 3.14	< 0.001	33.72 ± 3.76	< 0.001
MDS-UPDRS part 4c	8.78 ± 1.07	7.40 ± 0.81	< 0.001	6.82 ± 0.75	< 0.001
NMSS total	107.00 ± 21.86	100.78 ± 17.28	< 0.001	96.88 ± 16.11	< 0.001
PDQ-8 summary index	72.81 ± 8.82	58.44 ± 11.97	< 0.001	54.06 ± 12.28	< 0.001
HADS-depression	12.34 ± 1.86	10.38 ± 1.32	< 0.001	10.38 ± 1.32	n.s
HADS-anxiety	13.10 ± 1.96	10.32 ± 1.52	< 0.001	10.38 ± 1.32	n.s
PFS16	11.48 ± 3.35	9.66 ± 2.43	< 0.001	9.70 ± 3.18	< 0.001
MMSE	24.48 ± 2.90	24.48 ± 2.90	n.s	24.48 ± 2.82	n.s
PDSS	61.18 ± 19.18	66.10 ± 13.37	0.010	66.10 ± 13.37	n.s
King’s PD Pain Scale	54.67 ± 6.96	42.10 ± 7.25	< 0.001	39.92 ± 9.26	< 0.001

*LEDD*  levodopa equivalent daily dose, *MDS-UPDRS* Movement Disorder Society Unified Parkinson’s Disease Rating Scale, *NMSS* Nonmotor Symptoms Scale, *PDQ-8*  8-item Parkinson’s Disease Questionnaire, *HADS* Hospital Anxiety and Depression Scale, *PFS16* 16-item self-report Parkinson Fatigue Scale, *MMSE* Mini-Mental State Examination, *PDSS* Parkinson’s Disease Sleep Scale, *PD* Parkinson’s disease

^a^Wilcoxon test for baseline vs month 6 scores

^b^Wilcoxon test for baseline vs month 12 scores

Side effects were minor, all tolerated opicapone at 12 months and there were no dropouts. Side effects included mild dizziness, minor dyskiensias and nausea.

## Discussion

Our study evaluated the clinical effectiveness, safety and tolerability of third-generation COMT inhibitor opicapone in patients with PD on levodopa treatment at the 6 months and 12 months follow-up period in a non-White and Emirati community in UAE. A statistically significant improvement was observed among various motor and nonmotor domains, total levodopa intake and overall improvement in quality-of-life scores, suggesting that opicapone works well in this ethnic group even though there may be heterogeneity in the activity of the COMT enzyme, although this was not tested in our patients. Our study complements previous already established larger double-blind, randomized, placebo-controlled trials: BIPARK I (Ferreira et al. 2016), BIPARK II (Lees et al. 2017), OPTIPARK (Reichmann et al. 2020) and COMFORT-PD. Our study also complements and supports European and UK OPTIPARK study. Reichmann et al. (2020) showed statistically significant sustained improvement in both motor and nonmotor symptoms, especially in the pain, fatigue and sleep domains. The striking feature we report in our study is significant improvement in painful dyskinesias (MDS-UPDRS part 4), which often PD patients get after high/frequent levodopa dosing, along with improvements in pain score in the KPPS as a whole as well as overall nonmotor symptom burden (driven by pain, sleep, depression and anxiety benefits), quality of life scores as well as sleep, although the significant benefit in sleep was observed at 6 months and not at 12 months. Another impressive benefit was seen in the fatigue scale as well suggesting that augmenting levodopa effect by opicapone may also be beneficial for this disabling nonmotor symptom of PD. Our study findings are also in line with a previous OPEN-PD study (Tomlinson et al. 2010), which reported a significant reduction in total NMSS score during the follow-up with large effect in mood and sleep/fatigue domains, with sustained improvements at 6 and 12 months follow-up. As in OPTIPARK study (Reichmann et al. 2020) and other previous studies by Takeda et al. (Takeda et al. 2021) and Scott et al. (Scott 2021), we found significant improvement at 12 months follow-up for the NMSS score of fatigue, mood/apathy and hallucinations, and pain and smell.

Diversity in the use of recent anti-Parkinson medication is a major unmet need and has been the subject of much recent debate (Lau et al. 2022). Personalized medicine for Parkinson’s recommends that data on usage of drugs be reported in different communities, races and cultures, as responses may vary substantially owing to personality and cultural and pharmacogenetic issues (Titova and Chaudhuri 2017). Most of the data in relation to the use of opicapone as reported in BIPARK and OPTIPARK studies are based on White western Caucasian cohorts. Our study is one of the first to report the beneficial use of this drug in a non-White Emirati cohort and also signposts some specific effects such as improvement of painful dyskinesias as well as improvement in sleep and fatigue. We feel, therefore, that this study addresses a major gap in relation to the role of testing modern drugs in diverse populations and reporting of real-life effect moving away from a very selected largely White population that is often used in clinical trial settings.

The limitations of this study were the lack of a comparative arm and small sample size. Because of the sample size, a significant association may not be observed in some variables and specifically COMT activity was not tested in this cohort. Despite these limitations, the findings of this study are of great interest for clinical practice and consistently complements previous larger studies.

## Conclusion

Opicapone, a third-generation once-daily potent COMT inhibitor, provides excellent add-on or adjuvant effects spanning key motor symptoms as well as nonmotor symptoms of pain, fatigue and sleep, resulting in a significant benefit in the quality of life. In spite of racial differences, this study shows the sustained and significant benefit of opicapone use in a non-White and Emirati cohort for the first time.

## Data Availability

Raw data were generated at Kings College Hospital London, Dubai. Derived data supporting the findings of this study are available from the corresponding author VM on request.
